# The boon and bane of nitrous oxide

**DOI:** 10.1007/s00406-024-01801-3

**Published:** 2024-04-13

**Authors:** Golo Kronenberg, Georgios Schoretsanitis, Erich Seifritz, Sebastian Olbrich

**Affiliations:** 1 Department of Psychiatry, Psychotherapy, and Psychosomatics, University Hospital of Psychiatry Zürich, Lenggstrasse 31, P.O. Box 363, 8032 Zurich, Switzerland; 2 Department of Psychiatry Research, Zucker Hillside Hospital, Northwell Health, Glen Oaks, New York, USA; 3 Department of Psychiatry, Donald and Barbara Zucker School of Medicine at Northwell/Hofstra University, Hempstead, New York, USA

**Keywords:** Abuse, Depression, Homocysteine, Methylmalonic acid, Nitrous oxide, Rapid antidepressant effects

## Abstract

Nitrous oxide (N2O) has been known since the end of the eighteenth century. Today, N2O plays a huge role as a greenhouse gas and an ozone-depleting stratospheric molecule. The main sources of anthropogenic N2O emissions are agriculture, fuel combustion, wastewater treatment, and various industrial processes. By contrast, the contribution of medical N2O to the greenhouse effect appears to be small. The recreational and medical uses of N2O gradually diverged over time. N2O has analgesic and anesthetic effects, making it widely used in modern dentistry and surgery. New research has also begun studying N2O’s antidepressant actions. N-methyl-D-aspartate (NMDA) antagonism and opioid effects are believed to be the main underlying biochemical mechanisms. At this point, numerous questions remain open and, in particular, the conduct of larger clinical trials will be essential to confirm N2O’s use as a rapid-acting antidepressant. The N2O concentration delivered, the duration of a single inhalation, as well as the number of inhalations ultimately required, deserve to be better understood. Finally, the non-medical use of N2O has gained significant attention in recent years. Sudden deaths directly attributed to N2O are primarily due to asphyxia. Heavy, chronic N2O use may result in vitamin B12 deficiency, which, among other things, may cause megaloblastic anemia, venous thrombosis, myeloneuropathy, and skin pigmentation. Helpful biochemical tests include homocysteine and methylmalonic acid. The centerpiece of treatment is complete cessation of N2O use together with parenteral administration of vitamin B12.

## Introduction

**Fig. 1:**
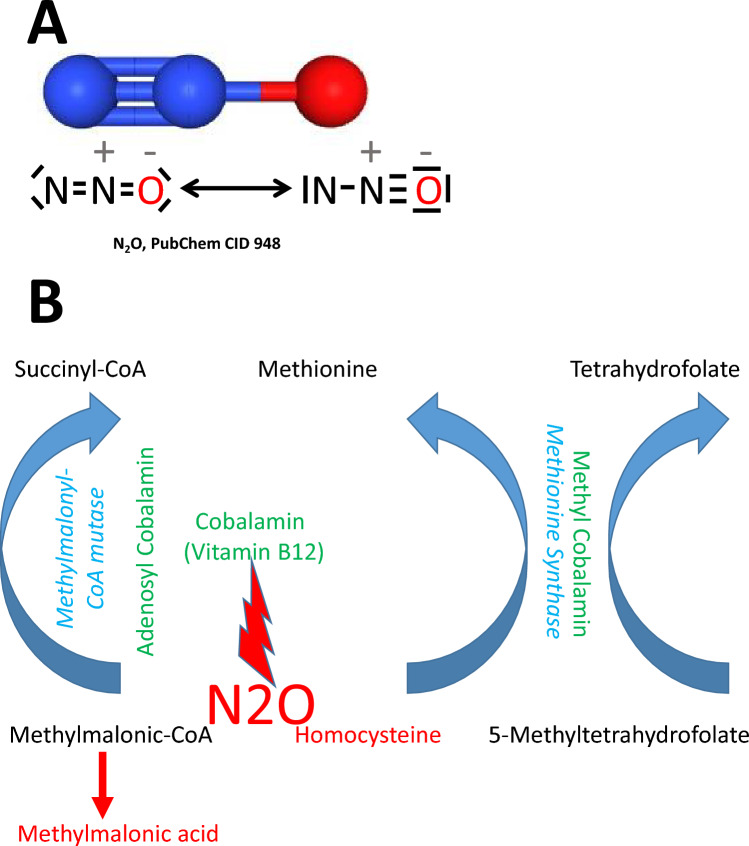
Nitrous oxide (N2O) and cobalamin (vitamin B12). **A** Chemical structure of nitrous oxide. **B** N2O inactivates vitamin B12 metabolic functions. Methylcobalamin is an essential coenzyme for methionine synthase which converts homocysteine to methionine and 5-methyltehtrahydrofolate to tetrahydrofolate. Adenosylcobalamin is an essential cofactor for methylmalonyl Co-A mutase which converts methylmalonyl-CoA to succinyl-CoA

Nitrous oxide is a colorless gas that has a slightly sweetish scent and a faintly metallic taste. The nitrous oxide molecule is more accurately described as dinitrogen monoxide. Its chemical formula is N2O. The N2O molecule has a linear asymmetric structure and a small electric dipole moment (Fig. 1A). Under room temperature and pressure, N2O is in the gaseous form (melting point: -91 °C; boiling temperature: -88 °C). The so-called critical temperature of N2O is 36.5 °C [20], meaning that, above this value, N2O will remain a gas irrespective of the pressure applied to it. Consequently, N2O may well be inhaled as a vapor, but it will definitely be exhaled as a gas.

Under normal conditions, N2O is stable and quite inert chemically. For this reason, N2O molecules remain in the atmosphere for about 121 years [24]. The majority of N2O is ultimately depleted in the stratosphere, where, nowadays, it acts as the single greatest ozone-depleting substance [43]. Next to carbon dioxide (CO2) and methane (CH4), N2O is also the third most important greenhouse gas. In effect, the impact of 1 pound of this frequently forgotten greenhouse villain on warming the atmosphere is approximately 270 times that of 1 pound of carbon dioxide [13]. Over thousands of years, atmospheric N2O concentrations rarely exceeded 280 parts per billion (ppb). However, levels have risen sharply since at least the 1920s, and, today, roughly 40% of the annual N2O emissions are accounted for by anthropogenic activities [24]. Besides agriculture, which exerts by far the largest effect, fossil fuels and industry also contribute very significantly to anthropogenic N2O emissions [46]. On the other hand, N2O from medical use is believed to contribute less than 0.05% to annual global greenhouse gas emissions [48].

## N2O as a medical gas: pharmacokinetics and pharmacodynamics

Today, N2O is produced on an industrial scale by thermal decomposition of ammonium nitrate at about 250 °C: NH4NO3 → 2 H2O + N2O. Impurities detected in medical cylinders of N2O are primarily due to nitrogen and oxygen. In the N2O cylinder, these two additional gases are mainly present in the initial gaseous form while the liquid phase underneath contains purer N2O [44]. Consequently, the N2O concentration actually increases over time as the cylinder empties [44].

The solubility of N2O is low in both blood and brain tissue [28]. N2O is therefore quickly absorbed and results in rapid onset and cessation of activity [5]. Barring the effects of bacteria in the gut, the only chemical reaction of N2O discovered in humans so far concerns cobalamin (i.e., vitamin B12), which N2O oxidizes irreversibly, thus inactivating it [40]. Cobalamin is a critical cofactor required by two hugely important enzymes (Fig. 1B), and the neurological and medical sequelae of N2O abuse seem to be driven primarily by inhibition of these two enzymes, i.e., methylmalonyl-CoA mutase and methionine synthase [33]. By impeding methylmalonyl-CoA mutase, N2O increases the concentration of methylmalonic acid. Similarly, inhibition of methionine synthase leads to elevated homocysteine levels (Fig. 1B). These two molecules can also be used as sensitive biomarkers for N2O toxicity [11]. Contrastingly, vitamin B12 deficiency caused by repeated or prolonged N2O exposure has been described as more difficult to detect. Indeed, vitamin B12 measured in blood frequently appears to be in the normal range even though the oxidized molecule (specifically, the oxidized cobalt atom) has become incapable of fulfilling its metabolic functions [31]. Concerning this point, it also seems important to stress the fact that administration of folate and vitamin B12 may not restore methionine synthase activity [42]. The reason is that, even when inactivated, cobalamin remains covalently bound to methionine synthase. In other words, it may take several days for the body to synthesize new methionine synthase with fresh, unoxidized cobalamin as an effective co-factor [42].

Applying histological and electrophysiological techniques using experimental rats, a landmark study from the Washington University School of Medicine demonstrated that N2O works chiefly as a strong *N*-methyl-d-aspartate (NMDA) antagonist but does not significantly inhibit γ-amino-butyric acid (GABA) gated currents [26]. In stark contrast, barbiturates such as pentobarbital and other inhalational agents such as halothane potentiate inhibitory synaptic GABA_A_ receptors [14]. Based on these findings, Nagele and colleagues subsequently performed in vivo experiments in Caenorhabditis elegans and found that N2O differs from volatile anesthetics such as halothane or isoflurane by producing different behavioral effects [37]. The behavioral alterations brought about by N2O were highly specific and characteristic of what was also produced by loss-of-function mutations in both NMDA and non-NMDA glutamate receptors [37]. Interestingly, akin to ketamine, another rapid antidepressant that has uniquely unequivocal uncompetitive inhibitory effects on NMDA receptors [53], N2O also enhances hippocampal excitatory transmission [25].

The second neuropharmacological mechanism widely associated with the effects of N2O is its opioid action [6, 34, 50]. Importantly, addictive properties of N2O have been linked with its effects on opioid receptors [17]. Moreover, competitive opioid antagonist naloxone appears to inhibit the analgesic effects of N2O [18]. Accumulating evidence also points to increased release of endogenous opioid peptides that apparently moderate at least some of the central nervous system effects produced by N2O [8, 9]. Interestingly, this set of facts once again brings to mind profound psychotropic analogies between N2O and ketamine, which show comparable strength of opioid action [16].

## The growing role of N2O until the middle of the twentieth century

Nitrous oxide was discovered and synthesized by English chemist, natural philosopher, and clergyman Joseph Priestley, who, among many other things, also discovered nitrogen and oxygen. In 1800, Sir Humphry Davy, a British chemist of the Pneumatic Institution in Bristol, published a monograph titled “Researches, Chemical and Philosophical; Chiefly Considering Nitrous Oxide”. He was the first to describe psychotropic effects of N2O such as “enjoyment”, “humor” and “laughter”, hence the lasting term “laughing gas”. The presumed anesthetic and analgesic properties of N2O were further investigated and introduced in dentistry and surgery around the middle of the nineteenth century by Horace Wells and William T. G. Morton.

The potential role of N2O in treating depression began to be investigated in earnest more than 100 years after its first discovery. In 1928, Julius Zádor from the University of Greifswald published his seminal work on the effects of N2O in psychiatry and neurology [52]. Unfortunately, he was not able to determine the precise amount of N2O and of oxygen administered in each case. At any rate, high concentrations of N2O were obviously used in all experiments for about 3 min. The psychiatric effects of the so-called “Lachgasrausch” (laughing gas rush) were investigated before, during, and shortly after inhalation [52]. Fifteen cases of depression, 36 cases of schizophrenia, and 34 mentally healthy participants were studied. In mentally healthy participants, the awakening phase frequently involved some form of cheerful excitement with laughter and talking. Regarding schizophrenia, Zádor felt that his study gave rise to scepticism about the benefits of N2O in this indication. Finally, regarding depression, he felt that N2O was more effective in patients suffering from “reactive” types of depression as compared to “endogenous” depression [52].

## Short meta-synthesis of the efficacy of N2O inhalation in treating depression

We performed a Medline search of all articles published in the English literature between 1955 and January 11, 2024, using the search terms “nitrous oxide” and “depression” or “depressive”. Table 1 summarizes the available randomized clinical trials (RCTs) of N2O in depression or treatment-resistant depression. For our subsequent analysis, the primary outcome was depressive symptoms at 24 h and one week post-treatment. Given the potential heterogeneity related to study populations, treatment procedures, and assessment methods, this analysis employed a random-effects model. Briefly, we estimated standardized mean differences (SMD) and 95% confidence intervals (95%CI) for ratings on the depression scales employed to assess the effects of N2O. We used the DerSimonian-Laird procedure for the estimation of the heterogeneity variance parameter (τ^2^) [12] and calculated the I-square (I^2^) statistic as an index of the variability potentially attributed to heterogeneity [21]. Additionally, we performed meta-regression analyses to assess the effects of age and sex. The meta package in R version 4.2.0 was used.

**Table 1:** Characteristics of the studies included in this review (in chronological order)

Author, year	Total subjects n	Diagnosis	Treatment resistance	Criteria for treatment-resistance	Study design	Group (N_2_O Dose)	n	Age (SD) In years	♀ (%)	Depressive symptoms assessment scales	Post-treatment change in depressive symptoms (mean, SD)				
											At 2 hours	At 24 h	At 1 week	At 2 weeks	At 4 weeks
Nagele, 2015	20	MDD (MINI)	√	Failure to ≥ 2 AD trials for current episode and ≥ 3 AD trials lifetime	Cross-over single dose	N_2_O (50%)	10^1^	44.3 (18.5)	12 (60.0)	HDRS-21	− 7.1 (7.6)	− 8.6 (6.8)	− 5.5 (7.6)	NA	NA
						Placebo	10^1^				− 2.9 (7.5)	− 4.7 (7.6)	− 4.4 (7.6)	NA	NA
Guimarães, 2021	51	MDD (DSM-5)	×	–	Double blind multiple dose	N_2_O (50%)	12	37.2 (13.6)	10 (83.0)	HDRS-17	NA	NA	NA	NA	− 16.7 (2.5)
						Placebo	11^2^	37.2 (12.8)	9 (82.0)		NA	NA	NA	NA	− 9.5 (3.3)
Nagele, 2021	24	MDD (MINI)	√	Failure to ≥ 1 AD trial for current episode and ≥ 3 AD trials lifetime	Cross-over single dose	N_2_O (25%)	20	44.3 (21.5)	17 (71.0)	HDRS-21	− 3.7 (1.0)	− 5.4 (1.4)	− 6.1 (1.4)	− 5.7 (1.5)	NA
						N_2_O (50%)	23				− 4.5 (4.4)	− 6.6 (6.5)	− 6.0 (7.1)	− 7.8 (7.1)	NA
						Placebo	22				− 2.7 (3.9)	− 3.6 (6.7)	− 2.6 (6.2)	− 0.1 (6.2)	NA
Yan, 2022	42	MDD (MINI)	√	Failure to ≥ 2 AD trials for current episode	Double blind single dose	N_2_O (50%)	20	34.0 (23–44)	9 (45.0)	HDRS-17	− 4.5 (7.4)	− 6.5 (6.4)	− 6.6 (7.5)	− 8.0 (10.7)	NA
						Placebo	22	28.0 (21–48)	14 (63.6)		− 1.4 (4.9)	− 3.4 (6.6)	− 5.5 (8.6)	− 6.6 (10.5)	NA
Kim 2023^3^	15	BD (SCID-IV)	√	Duration of current episode^3^	Double blind single dose	N_2_O (25%)	12	33.6 (7.1)	6 (50.0)	MADRS	NA	− 16.2 (6.1)	NA	NA	NA
						Midazolam	13	34.6 (9.7)	7 (54.0)		NA	− 14.6 (7.1)	NA	NA	NA

♀ females, *AD* antidepressants, *BD* bipolar depression, *DSM* Diagnostic and Statistical Manual of Mental Disorders, *HDRS* Hamilton Depression Rating Scale, *MADRS* Montgomery–Åsberg Depression Rating Scale, *MDD* major depressive disorder, *MINI* Mini Neuropsychiatric International Interview, *NA* not available, n number of patients, *SCID* Structured Clinical Interview for DSM-IV, *SD* standard deviation

^1^Response data refer to first treatment session

^2^Two patients were not considered in the final analysis

^3^Duration of current episode was 3.6 ± 4.7 vs. 5.9 ± 9.6 years in the N_2_O and placebo groups, respectively

**Fig. 2:**
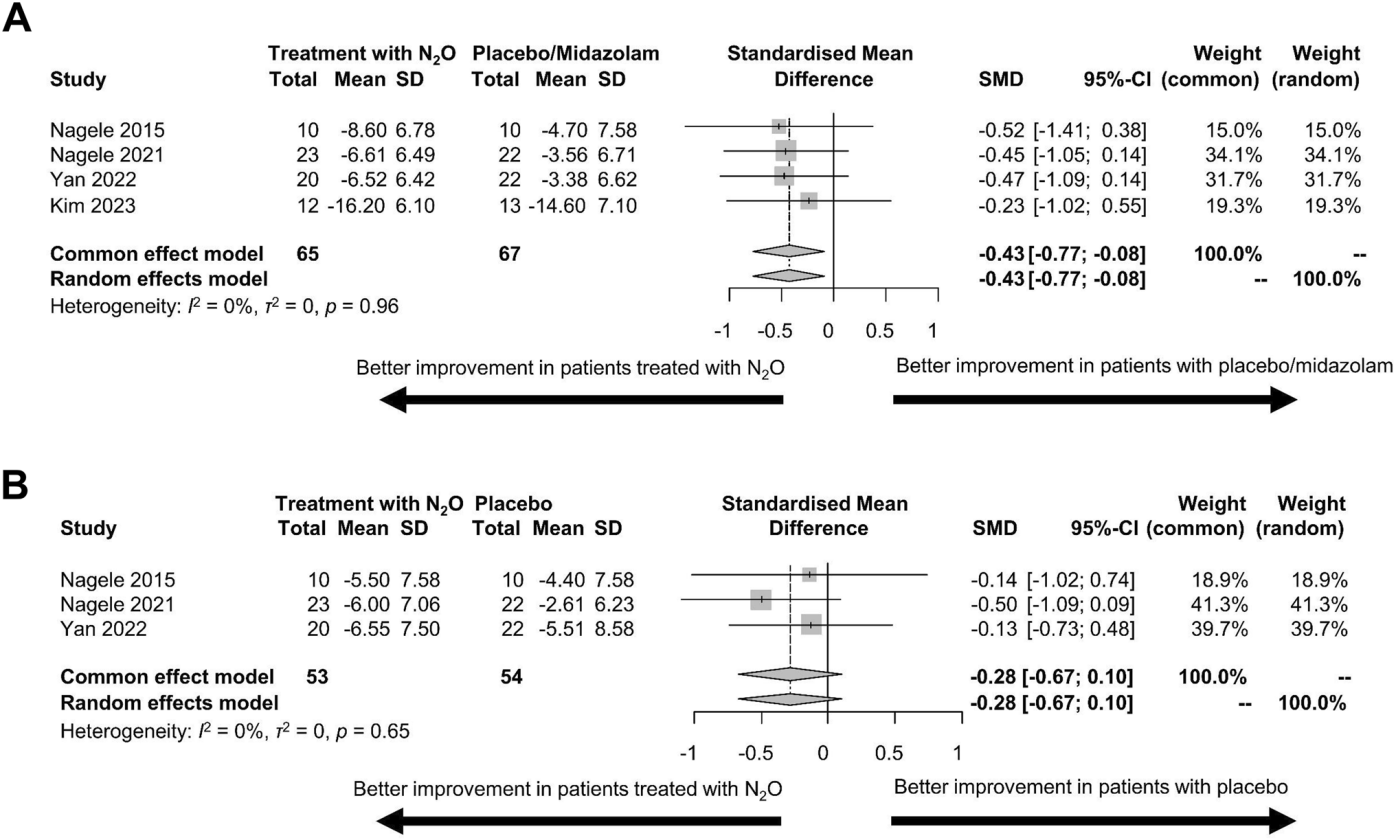
Meta-synthesis of the available literature on inhalation of N2O for treating depression.** A** Changes in depressive symptoms in patients treated with N2O compared to placebo/midazolam at 24 h post-treatment. **B** Changes in depressive symptoms in patients treated with N2O compared to placebo at one week post-treatment. *CI* confidence interval, *N2O* nitrous oxide, *SD* standard deviation, *SMD* standardized mean difference

In short, we included four studies [29, 36, 38, 49] investigating 101 depressed patients in our meta-analytical sample (mean age, 36.9 ± 14.0; n = 65 females). One study used a multiple-dose design with a longer follow-up [19] compared to the remainder of the studies and was therefore not included (Table 1). Concerning the primary outcome, we only considered the N2O concentration of 50% from the study by Nagele and co-workers that included two N2O concentrations (25% and 50%) [38], as the majority of the available studies applied the concentration of 50%. At 24 h post-treatment, four trials suggested a larger decrease in depressive symptoms in patients receiving N2O compared to placebo/midazolam (SMD = − 0.43, 95% CI = − 0.77 to − 0.08, p = 0.02, Fig. 2A). Heterogeneity was minimal (*I*^2^ = 0.0%, *τ*^*2*^ < 0.001). At one week post-treatment, three trials suggested a larger decrease in depressive symptoms in patients receiving N2O compared to placebo. However, this difference was not statistically significant (SMD = -0.28, 95% CI = − 0.67 to 0.10, p = 0.15, Fig. 2B). Again, heterogeneity was minimal (*I*^2^ = 0.0%, *τ*^*2*^ < 0.001). Finally, in our meta-regression analyses, age did not have an impact on changes in depressive symptoms at 24 h (estimated co-efficient 0.01, 95%CI = − 0.04 to 0.04, p = 1.00) or one week post-treatment (estimated co-efficient 0.02, 95%CI = − 0.02 to 0.05, p = 0.42). Similarly, we did not detect an effect of the percentage of females on changes in depressive symptoms (estimated co-efficient − 0.01, 95% CI = − 0.05 to 0.04, p = 0.81 at 24 h and -0.02, 95% CI = − 0.08 to 0.03, p = 0.36 at one week post-treatment, respectively).

To sum up, there are, even today, only a handful of clinical trials that have studied N2O as a new treatment option for depression. Several points merit special attention. First, the overall number of study participants reported in the literature remains low (Table 1). Moreover, the number of patients reported in each individual study appears small, even in studies recently published in high-impact journals. Therefore, generally speaking, the obtained results are highly interesting and encouraging, but should be interpreted with a certain amount of caution until replicated in larger samples. Second, both placebo control and blinding are much harder to perform when using inhalation of N2O as compared to a conventional oral antidepressant. Based on earlier research, it seems at least plausible that quite a number of patients were able to differentiate between inhalation of N2O and placebo [7, 15]. Strikingly, the most recent study listed in Table 1 [29] is the only study that deployed a more active placebo (i.e., comparison of 25% N2O plus intravenous saline versus inhalation of medical air plus 2 mg intravenous midazolam). It is also the only clinical trial published so far that yielded a negative primary outcome (no significant between-group differences in 24-h post-treatment Montgomery–Åsberg Depression Rating Scale change or treatment response; Table 1). Third, it should also be noted that the putative duration of N2O’s antidepressant effects ranges widely from less than 24 h to more than 2 weeks (Table 1). A recent case report even described remission of major depressive disorder after single nitrous oxide inhalation for more than a month [35].

Taken together, the use of N2O in psychiatry seems auspicious at a time when new treatments for major depression are finally emerging. In certain patient populations, off-label N2O inhalation should, even today, be considered seriously as a promising novel treatment option. Two factors are especially important: (1) N2O appears as a putative rapid-acting antidepressant. (2) Based on the available literature, it seems unlikely that a single inhalation of N2O will ultimately suffice in most patients. Especially when multiple inhalations are performed, exams and checkups may be helpful. Contraindications for N2O should be taken seriously. They include critical illness, severe cardiac disease, pulmonary hypertension, pneumothorax, and the first trimester of pregnancy [30]. As described above, vitamin B12 administration may not completely reverse N2O-induced metabolic changes, so measurement of plasma homocysteine levels may be advisable before and during the course of repeated N2O inhalations.

## Abuse and neurotoxicity

Recreational N2O use first developed in the British upper classes more than 200 years ago. The young Robert Southey, the future Poet Laureate, wrote to a friend in 1799, “*Such a gas has Davy discovered! The gazeous oxyd! Oh Tom! I have had some. It made me laughed and tingled in every toe and fingertip. Davy has actually invented a new pleasure for which language has no name. Oh Tom! I am going for more this evening—it makes one strong and so happy! So gloriously happy!*”

The exact prevalence of recreational N2O use remains at least partially unknown. According to the Office of National Statistics, approximately 444,000 individuals engaged in non-medical use of N2O in England and Wales in 2022 [41]. The prevalence of N2O use was around three times higher in adults aged 16–24 years than in adults aged 16–59 years (3.9% and 1.3%, respectively; [41]).

In a nutshell, today, non-medical N2O use, although not studied systematically, still appears to occur primarily in younger people. “Whippets” or “nangs”, i.e., small N2O cartrages, frequently belong to a certain youth subculture and have gained notoriety at nightclubs, parties, and dorm rooms. Here, the psychotropic effects of N2O abuse usually occur much more quickly and briefly, but typically also result from higher immediate concentrations, than those currently associated with antidepressant treatment (i.e., mostly 60 min; [19, 36, 38, 49]). Heavy, regular N2O use appears relatively uncommon. It is believed to be the main reason for neurotoxicity. Based on the Diagnostic and Statistical Manual of Mental Disorders, Fifth Edition (DSM-5) criteria for substance use disorder [1], the scant literature suggests that N2O addiction may indeed exist in the heaviest N2O users [2].

On the whole, the number of deaths straightforwardly attributed to recreational N2O use still appears low. Asphyxia is a critical risk. Particularly in closed spaces, N2O may quickly displace environmental oxygen [47]. These high-profile cases that often occur on college campuses or in relation to younger people are regularly covered in the media. In addition, in recent years, a spate of dramatic car crashes and fatalities (including bystanders) has further raised political awareness in many countries. As a consequence, as of 8 November 2023, it became illegal in the United Kingdom “*to possess, supply, import, export or produce nitrous oxide outside of its intended purposes*” [22]. It remains to be seen how this and similar legal changes will ultimately affect the damage and prevalence of N2O abuse, toxicity, and associated deaths.

A single inhalation of N2O does not usually cause neurotoxic changes unless the level of vitamin B12 had already been too low. Briefly, elevated homocysteine levels (Fig. 1B) may cause thrombophilia and venous thrombosis following a history of N2O inhalation [3]. Similarly, reduced methionine levels (Fig. 1B) result in reduced S-adenosylmethionine, which, in turn, impacts DNA, RNA and protein metabolism [10]. Consequently, among other things, megaloblastic anemia [4] and myeloneuropathy may occur [39]. From a clinical perspective, common symptoms include paresthesia and gait unsteadiness [45]. Additional symptoms frequently include bladder and bowel disturbances [32]. Skin pigmentation may occur [51]. In these cases, chronic N2O abuse should always be considered, especially when younger patients are involved. Helpful biochemical tests include homocysteine and methylmalonic acid levels as well as mean corpuscular volume, all of which may be increased [32]. Subacute combined degeneration is frequently observed on MRI [27]. The centerpiece of treatment is complete cessation of N2O use. In addition, parenteral administration of vitamin B12 should be performed quickly (typically 1000 μg/day for 5 days followed by oral vitamin B12 for several months) [23].
